# Relationship between care-givers' misconceptions and non-use of ITNs by under-five Nigerian children

**DOI:** 10.1186/1475-2875-10-170

**Published:** 2011-06-22

**Authors:** Ekundayo D Arogundade, Samson B Adebayo, Jennifer Anyanti, Ernest Nwokolo, Olaronke Ladipo, Augustine Ankomah, Martin M Meremikwu

**Affiliations:** 1 Society for Family Health, Port Harcourt Crescent, Area 11, Abuja, Nigeria; 2 Department of Paediatrics, University of Calabar, Calabar, Nigeria

## Abstract

**Background:**

Malaria has been a major public health problem in Nigeria and many other sub-Saharan African countries. Insecticide-treated nets have shown to be cost-effective in the prevention of malaria, but the number of people that actually use these nets has remained generally low. Studies that explore the determinants of use of ITN are desirable.

**Methods:**

Structured questionnaires based on thematic areas were administered by trained interviewers to 7,223 care-givers of under-five children selected from all the six geo-political zones of Nigeria. Bivariate analysis and multinomial logit model were used to identify possible determinants of use of ITN.

**Results:**

Bivariate analysis showed that under-five children whose care-givers had some misconceptions about causes and prevention of malaria were significantly less likely to use ITN even though the household may own a net (p < 0.0001). Education and correct knowledge about modes of prevention of malaria, knowing that malaria is dangerous and malaria can kill were also significantly associated with use of ITN (p < 0.0001). Knowledge of symptoms of malaria did not influence use of ITN. Association of non-use of ITN with misconceptions about prevention of malaria persisted with logistic regression (Odds ratio 0.847; 95% CI 0.747 to 0.960).

**Conclusions:**

Misconceptions about causes and prevention of malaria by caregivers adversely influence the use ITN by under-five children. Appropriate communication strategies should correct these misconceptions.

## Background

Everyone living in all parts of Nigeria is at risk of malaria infection. *Plasmodium falciparum *accounts for 90-95% of malaria infections in Nigeria. Transmission of malaria occurs throughout the year with the intensity higher in the southern parts of the country because of the longer rainy season that favours the breeding of mosquitoes. The species of mosquitoes that commonly transmit malaria in Nigeria are *Anopheles gambiae, Anopheles funestus *and *Anopheles arabiensis *[1].

Malaria attacks are more frequent in young children aged below five years who lack the protective immunity, and are therefore more likely to become suffer from severe malaria and to die from the disease. Severe malaria in young children could present with such life-threatening complications as severe anaemia, respiratory distress, repeated convulsions and unconsciousness. Severe malaria can develop from uncomplicated malaria in a few hours, especially if the malaria has not been properly treated or is caused by a drug-resistant parasite. Severe malaria accounts for a third of all childhood deaths in Nigeria [2]. Children who survive cerebral malaria may develop neurological abnormalities like deafness, blindness, speech disorders, epilepsy and learning disabilities which persist in a few children with significant impairment in their development and education [3].

The use of insecticide-treated nets is currently considered one of the most cost-effective methods of malaria prevention in highly endemic areas. The use of insecticide-treated meets (ITNs) or long-lasting insecticidal nets (LLINs) is the main method of malaria prevention employed in Nigeria. Free distribution of long lasting insecticidal nets (ITNs) is conducted through campaigns, public health facilities, faith-based organisations (FBO), and non-governmental organisations (NGOs) with the goal of achieving universal access for the at- risk population of children under age five and pregnant women[2].

Regular use of insecticide-treated nets by all those at risk of malaria infection is a key component of the national malaria control strategy. Controlled community trials conducted across different epidemiological settings provide strong evidence on the effectiveness of insecticide-treated mosquito nets against malaria infections resulting in marked reduction of all-cause mortality among preschool children in most trials [4]. Regular use of ITNs by pregnant women has also been shown to reduce the risk of maternal anaemia, placental malaria, low birth weight and perinatal mortality [5]. Despite these well-known benefits of ITNs and the efforts of the Nigerian government to promote this intervention, many families and individuals at risk in the country do not own or use them [6]. Several national surveys have shown persistently low levels of ownership and use of insecticide-treated nets. The proportion of Nigerian households that owned at least one insecticide-treated nets (ITN) was 2.2% in 2003 and 8% in 2008 while the proportion of under-five children that used ITN during the same periods were 1.2% and 5.5% respectively [7,8].

Poor perception and knowledge of malaria control is among the factors likely to influence use of ITNs. In south-eastern Nigeria, excessive heat was thought to be responsible for malaria [9]. Among the perceived causes of malaria in south-western Nigeria, were over-work, sunlight, excessive sex and noise as well as witchcraft [10]. Such erroneous perception of malaria as "ordinary fever" that is caused by "too much work" or "too much sun" have been reported across a wide range of cultural sub-groups in Nigeria, and believed to significantly influence treatment-seeking behaviour and attitude to preventive measure [11]. Caregivers' perception of malaria as simple or ordinary fever may adversely influence the choice of treatment or delay the time to an appropriate treatment action [12]. Identifying such misconceptions for the purpose of designing appropriate educational interventions could lead to improvement in treatment-seeking and preventive practices as shown by the report of an educational intervention in Northern Nigeria [13].

The need to understand perceptions and practices of the community about malaria has been emphasized as vital ingredient in the design of communication strategies for educational interventions. Currently, the Nigerian malaria control programme emphasizes the behavioural change communication strategies as an integral part of the mass ITN distribution campaigns [2]. Understanding the local perceptions and practices could be of immense relevance to such interventions that seek to enhance community's potential to adopt and sustain the use of ITNs. The analysis of information on caregivers' perception and knowledge of malaria against the prevailing rate of utilisation of insecticide-treated nets is presented in this paper with a view to highlight magnitude of existing knowledge gaps and the implications this may have for increasing and sustaining the use of ITNs.

## Methods

### Study area and population

Twenty one States (including the Federal Capital Territory) out of the 36 States that make up Nigeria were included in this study. Malaria control in 18 of these states was supported by the Global Fund grants while three were supported by funds from other international development partners. Selection of these states for intervention was based on the burden of the disease and existence of implementing partners currently working on prevention of malaria. All the states were implementing programmes that draw from the national strategic plan, which is based on the principles of the global malaria control strategy and Roll Back Malaria. In this paper, the study population was based on a representative sample of caregivers of children under the age of 5 years in these states. The study is a population-based cross-section survey. Probability sampling design was used. Multi-stage cluster sampling design was used to select samples of caregivers of children under five years of age. The selection process aimed at achieving a representative sample of study participants spread across each state in such way as to reflect the social, economic, and rural urban distribution of the population in the state.

### Survey procedures

Given that the objective of the survey is to measure the malaria-related maternal and child care indicators in the States where the survey was conducted, the analysis was designed to be run at state level thereby making the state the reporting domain and the basis for calculation of the sample size. Survey sample size was therefore calculated with a view to making the size of data from each state adequate for state-level interpretation of results. Since the proportion of children (age below 5) that sleep under mosquito net for each of the states was among the indicators measured in the study, this was used as a benchmark for sample size calculation. Selection was based on a stratified probability sampling technique based on locality of residence (i.e. rural and urban). Individual respondents were selected within households in various enumeration areas across the country. Within a state (administrative division), all eligible persons (i.e. caregivers) irrespective of the nature of residence (rural or urban) were given equal chance of being included in the final sample. Using appropriate formula for detecting a minimum change of 10 percent between two time points (i.e. baseline and follow up), 5 percent level of significance and 80 percent power of statistical test, the calculated sample size was at least 346 per State allowing for non-response rate of 10%. Overall, a minimum sample of 346 households with at least one child aged under five years was targeted per state. A sampling frame of enumeration areas in those states was based on the 2006 Population Census figures [14]. Data were collected from caregivers of children less than five. Trained interviewers visited each of the selected localities. The quota allocated to each locality was identified through systematic random sampling and probability proportionate to size.

Structured questionnaires based on thematic areas were administered by trained interviewers. The questionnaire was validated and pilot-tested before use. Interviewers were trained with a specific focus on all aspects of the interview process and questionnaires. The questionnaires were pre-tested to check for comprehensibility of questions as well as procedures for conducting interviews. Questionnaires were primarily in English due to the linguistic complexity of Nigeria of the states where the surveys took place, but in each state, key words/phrases were translated. Interviewers were selected based on their understanding of both English and the local languages of the communities where they worked. The study questionnaires elicited information on key programme indicators and the various issues related to prevention and treatment of malaria in the target groups. Specifically, the questionnaire had a screening question to ensure only household with children under five were interviewed also it elicited information about respondents' background characteristics, knowledge of malaria: causes, prevention and treatment, use of long lasting insecticide treated net, reported incidence of malaria, knowledge of ACT and media exposure to behaviour change communication on malaria control. The study questionnaire has been submitted as an additional file 1.

Approval was received from the leaders of each of the communities selected for the survey. Informed verbal consent was obtained from the heads of households and caregivers involved in the interview; those who declined consent were not interviewed. All selected caregivers were interviewed in their homes and confidentiality was assured. During data collection, quality control processes were implemented by supervisors to identify and correct problems while in the field. Possible determinants of use of ITN and antenatal visits were explored in addition to other background characteristics of the respondents.

### Statistical procedures

Data were entered using the Census Surveys Professional software, and was exported to, managed and analysed using SPSS 18.0^®^. Frequency tables and cross tabulations were used to generate data Tables and Figures to present results in keeping with survey objectives and analysis plan. The key dependent variable in this paper is 'ownership and use of a bed net'. A logistic regression technique was employed to explore influence of factors, such as misconception about causes and prevention of malaria, knowledge about ITN, causes of malaria and correct ways of preventing malaria on use of ITNs. At an exploratory data analysis stage, several models were explored. Specifically, exposure to SFH mass media on misconceptions about malaria was explored with the aim of being able to attribute lower levels of misconceptions to exposure to various mass media campaigns. All categorical variables were dummy-coded. A binary dependent variable was created for use or non-use of ITN and determinants. Statistical significance was based on a p-value of 0.05. Test of goodness-of-fit was based on Hosmer and Lemeshow test (HL); with models with p > 0.05 assumed to be good and fit well to the data.

#### Dependent variable

The dependent variable of interest in this paper is the use of ITN by children under 5. In this study, it was difficult to differentiate between long-lasting insecticide-treated nets and retreated nets. Therefore, the outcomes were combined. Thus, in this paper, all insecticide-treated nets, whether they were long-lasting insecticide-treated nets or retreated nets, will be referred to subsequently as ITNs. In this paper, a three-level nominal categorical outcome variable describing ownership and use of ITNs was created. The rationale behind this was to elicit information about different categories of ownership and use, ownership but do not use, and don't own a net. Through this, one can identify determinants of each of the categories described above.

#### Independent variables

The key independent variables in this paper are: misconception about causes and prevention of malaria, geo-political zones, educational attainment, knowledge about use of ITN, locality of residence (rural or urban), knowledge about modes of prevention of malaria, age of the respondents, and knowledge that mosquito bites is the main cause of malaria. All these independent variables were dummy-coded. For instance, five dummies were created for geo-political zones (North East, North West, South East, South West and South South) with North Central being the reference category. For locality of residence, rural was considered as reference, for age category 30 years and above was considered as reference category. Education was an ordinal variable, with four possible categories regarding the highest level of education attained: no formal education, primary, secondary, and post-secondary education. Nigeria was divided into six geographic zones; each zone comprising of 3-4 contiguous states. North central was assumed as the reference category.

### Bivariate analyses

The primary outcome variable in this paper was based on three level categorical outcomes for ownership and use of insecticide-treated or long-lasting insecticide net. Possible association between the outcome variables and other determinants of net-use were explored using a Pearson chi-square statistic for contingency tables.

### Multivariate analyses

In this paper, the dependent variables were created based on ownership and use of a treated or long-lasting insecticidal bed net. This variable was based on a three level categorical outcome defined as(1)

Through this, analysis will permit us to understand and discern the determinants of two of the categories of the outcome variable - ownership and use of ITNs while one category is considered as the reference category for comparison. In this case study, the third category was used as the comparison category (i.e. reference category is the respondents who do not own a net or no response). Modelling possible association between misconception about malaria and the outcome variable y_1 _in the presence of other determinants of net-use requires a regression approach that considers the exhaustive categories of the dependent variable. In literature, common statistical technique for handling such situation includes either a multinomial probit or multinomial logit model (see [15,16]). However, for ease of interpretation through odds-ratio, a multinomial logistic regression model was employed in this paper for modelling this categorical outcome.

## Results

### Biosocial characteristics and descriptive analysis

**Figure 1:**
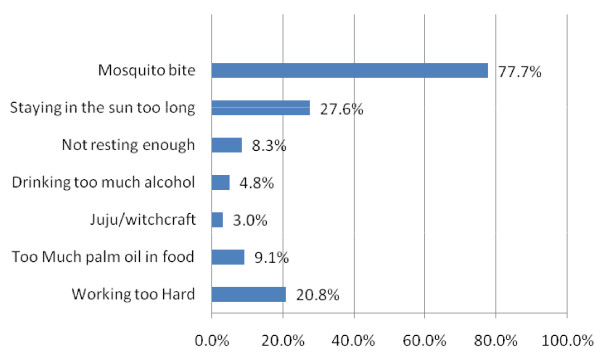
**Caregivers' perception of the cause(s) of malaria**.

**Figure 2:**
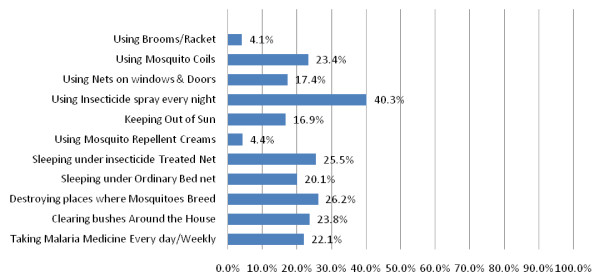
**Caregivers' knowledge of how to prevent malaria**.

Table 1 presents biosocial characteristics and findings of the descriptive analysis of the caregivers interviewed in this survey. Forty four (44) percent of the respondents resided in urban areas while 46% lived in rural areas. Most caregivers are in the age range 30 years and above. The overall mean age of caregivers was 31.1 years with a standard deviation of 9.23 years. Respondents with higher education in urban areas are more than twice of their counterparts in rural areas. Majority of the respondents have secondary education. On the knowledge of causes of malaria, more than three-quarters of the caregivers knew that mosquito bites could cause malaria. Figure 1 presents caregivers' perceptions about causes of malaria. Figure 2 presents the caregivers' knowledge and misconceptions about how to prevent malaria. Only about 25 percent of the caregivers mentioned sleeping under ITNs as a means of preventing malaria. Using insecticide sprays every night was the most commonly mentioned method of preventing malaria (40%).

**Table 1:** Percentage distribution of respondents' socio-demographic characteristics

	% Frequency/Mean(SD)		
	
Characteristics	Urban	Rural	Total/Mean(SD)
**Age of caregivers**			
15 - 19	4.8	7.9	421
20 - 24	16.8	18.5	1155
25 - 29	26.9	23.3	1629
30 and above	51.4	50.2	3308
Age continuous: mean (SD)	31.29 (8.93)	30.95 (9.47)	31.11(9.23)
**Education**			
None	14.3	27.2	1533
Qur'anic only	10.2	15.7	951
Primary	20.1	23.3	1575
Secondary	41.1	26.9	2419
Higher	14.2	6.9	745
**Geopolitical Zones**			
North West	16.4	19.7	1313
North East	17.3	22.5	1453
North Central	18.5	19.6	1379
South West	22.2	8.5	1072
South East	11.1	14.9	946
South South	14.5	14.8	1060
**Knowledge of symptoms of malaria**			
Hotness of the body/high temperature	97.5	98.0	7062
Dark coloured urine	83.3	85.0	6081
Joint pains	81.5	83.4	5959
Headache	90.8	92.5	6625
Loss of appetite	89.5	90.0	6485
Vomiting	79.6	81.2	5810
Weakness/dizziness	83.8	86.3	6148
Talking nonsense/disorientation	41.8	47.2	3226
Difficulty in breathing	41.1	49.7	3301
Coma	33.8	38.1	2605
Persistent vomiting/vomiting everything	47.7	50.5	3552

Results of bivariate analysis are presented in Table 2. Educational attainment of caregivers, geopolitical zones, knowledge of mosquito bites as a cause of malaria, knowledge of prevention of malaria, misconception about causes of malaria and misconception about prevention of malaria were significantly associated with use of ITN/LLIN (p < 0.05). A significant positive association was observed for education with caregivers who have higher level of education most likely to own and use a net compared with caregivers with no education (p < 0.0001). Also those in the North East of Nigeria are most likely to own and use a net compared with their counterparts in other geopolitical zones (p < 0.0001).

**Table 2:** Bivariate analysis of knowledge and misconception about causes and prevention of malaria according to selected socio-demographic characteristics

Characteristics	Three categorical outcomes						Total studied
	Used ITN	Own but did not use ITN	Do not own	P-values	**X**^**2**^	**d.f**,	
**Locality of residence**				0.962	0.08	2	
Rural	27.2	5.9	66.9				3867
Urban	27.4	6.0	66.6				3356
**Age of caregivers**				0.100	10.63	6	
15 - 19	29.5	5.7	64.5				421
20 - 24	26.8	6.1	67.1				1155
25 - 29	29.6	7.2	63.2				1629
30 and above	27.1	5.7	67.3				3308
**Education**				<0.0001	95.02	8	
None	23.9	3.2	72.9				1533
Qur'anic only	28.1	6.4	65.5				951
Primary	25.0	6.9	68.2				15752
Secondary	27.4	6.4	66.2				2419
Higher	37.9	7.9	54.2				745
**Geopolitical Zones**				<0.0001	132.89	10	
North West	28.8	7.3	63.9				1313
North East	37.7	3.7	58.6				1453
North Central	25.0	5.9	69.1				1379
South West	21.2	5.7	73.1				1072
South East	24.7	6.7	68.6				946
South South	22.6	7.2	70.2				1060
**Knowledge Hotness of body/high temperature**				0.276	2.58	2	
Yes	27.4	6.0	66.6				7062
No	21.7	6.2	72.0				161
**Knowledge of mosquito bites**				<0.0001	31.20	2	
Yes	28.8	5.5	65.7				5601
No	22.3	7.5	70.2				1611
**Correct knowledge of prevention of malaria**				<0.0001	45.32	2	
Yes	29.6	5.7	64.7				4991
No	22.0	6.6	71.3				2232
**Misconception about causes of malaria**				<0.0001	37.09	2	
Yes	24.5	6.1	69.4				4081
No	30.9	5.8	63.3				3142
**Misconception about malaria prevention**				<0.0001	32.46	2	
Yes	24.8	6.1	69.2				4205
No	30.8	5.8	63.4				3018

Nearly three in ten of the caregivers that knew that mosquito bite is a cause of malaria, and knew the correct ways of preventing malaria owned and used a net (p < 0.05). On the issue of misconception about causes and prevention of malaria, almost one quarter of the respondents who did not have any misconception (either about causes or prevention of malaria) owned and used a net. In each case, respondents were asked about causes of malaria and how one can prevent malaria. A respondent holds a misconception if he/she mentions what is not a known cause of malaria or what is not a known means of preventing malaria. All these misconceptions can be considered as non-biological causes of malaria. For instance, of the misconception mentioned were: not resting enough or lack of sleep, drinking too much alcohol/beer, witchcraft/juju, eating too much palm/ground nut oil and working too hard/stresses.

Three separate multivariate models were fitted and results of these are presented in Table 3. At a first stage, a multinomial logistic model of y on misconceptions about causes and prevention of malaria was fitted. Model 2 adjusts for caregivers' knowledge about malaria such as knowledge of symptoms of malaria, causes of malaria, prevention of malaria, that malaria is dangerous, and that malaria can kill. In model 3, other determinants of use of ITN such as age, educational attainment level, locality of residence, and geopolitical zones were adjusted for. Good of-fit test was based on Likelihood Ratio (LR) statistics that follow a Chi-square distribution with *k *degrees of freedom (d.f.). The LR for the fitted models are 50.46 (*n *= 7223, *d.f *= 4, *p *< 0.0001), 259.13 (*n *= 6513, *d.f*. = 30, *p *< 0.0001) and 290.29 (*n *= 6504, *d.f*. = 40, *p *< 0.0001) for models 1, 2 and 3 respectively.

**Table 3:** Parameter estimates and corresponding p-values for models 1, 2 and 3 logistic regression (including 95% confidence intervals for model 3)

Variables	Model 1 (n = 7223)		Model 2 (n = 6513)		Model 3 (n = 6504)			
	
	Odds Ratio	P-value	Odds Ratio	P-value	Odds Ratio	P-value	Confidence	Interval
							
							Lower	Upper
**Own and use a net**								
Misconception about prevention of malaria	0.807	<0.0001	0.851	0.007	0.847	0.009	0.747	0.960
Misconception about causes of malaria	0.783	<0.0001	0.798	<0.0001	0.888	0.074	0.779	1.012
*Knowledge of*								
Symptoms of malaria			1.241	0.273	1.169	0.471	0.764	1.790
Causes of malaria			1.083	0.305	1.134	0.138	0.960	1.338
Malaria prevention			1.330	<0.0001	1.181	0.028	1.018	1.370
Malaria is dangerous			2.643	0.011	1.798	0.133	0.837	3.864
Malaria can kill			2.792	0.007	2.047	0.067	0.952	4.404
*Age*								
15 to 19 years					1.146	0.250	0.908	1.447
20 to 24 years					1.018	0.827	0.869	1.192
25 to 29 years					1.174	0.021	1.024	1.346
*Educational attainment*								
Qur'anic					1.078	0.485	0.872	1.333
Primary					1.448	<0.0001	1.201	1.744
Secondary					1.773	<0.0001	1.484	2.119
Higher					2.642	<0.0001	2.127	3.281
Urban					0.944	0.341	0.840	1.062
*Geopolitical zones*								
North West					1.227	0.052	0.998	1.509
North East					2.037	<0.0001	1.699	2.441
South West					0.999	0.993	0.807	1.237
South East					1.064	0.555	0.866	1.307
South South					0.932	0.499	0.759	1.144
**Own a net but did not use**								
Misconception about prevention of malaria	0.958	0.694	0.964	0.743	0.981	0.875	0.775	1.243
Misconception about causes of malaria	0.986	0.898	0.919	0.460	0.928	0.552	0.726	1.187
*Knowledge of*								
Symptoms of malaria			1.073	0.833	1.404	0.428	0.607	3.248
Causes of malaria			0.743	0.024	0.830	0.187	0.630	1.094
Malaria prevention			1.046	0.715	0.991	0.946	0.765	1.284
Malaria is dangerous			1.866	0.288	4.320	0.150	0.589	1.284
Malaria can kill			1.680	0.386	3.622	0.206	0.493	26.614
*Age*								
15 to 19 years					1.067	0.777	0.680	1.675
20 to 24 years					1.107	0.492	0.828	1.481
25 to 29 years					1.295	0.039	1.013	1.656
*Educational attainment*								
Qur'anic					1.840	0.008	1.176	2.878
Primary					2.517	<0.0001	1.707	3.712
Secondary					2.319	<0.0001	1.582	3.399
Higher					3.549	<0.0001	2.285	5.510
Urban					0.943	0.594	0.759	1.171
*Geopolitical zones*								
North West					1.360	0.100	0.943	1.961
North East					0.816	0.292	0.559	1.191
South West					0.923	0.674	0.636	1.340
South East					1.061	0.749	0.740	1.520
South South					1.078	0.670	0.765	1.518

Table 3 presents the results of regression from the multinomial logit outcome y. In the three models, caregivers with some misconceptions either about prevention or causes of malaria are less likely to use net compared with those who do not own a net. For instance, caregivers with misconceptions about prevention of malaria are about 20 percent less likely to own and use a net (OR = 0.81, p < 0.0001). Similarly caregivers with some misconception about causes of malaria are about 25 percent less likely to own and use a net. In this case, misconceptions about causes and prevention of malaria are risk factors for malaria. However, the net-effect of net use for misconception about causes of malaria declines as further determinants of use of ITN were controlled for in model 3. Thus the effect of misconception about causes of malaria becomes insignificant in model 3. Further discussions of results are based on findings from model 3. Caregivers with correct knowledge of prevention of malaria are about 18 percent more likely to own and use a net compared with their counterparts who do not own a net (OR = 1.18, p = 0.028). This was significant with p = 0.028. In this case, correct knowledge of preventing malaria is a protective effect. Caregiver's age is positively associated with ownership and use of ITN. Respondents in age group 25 to 29 years are about 17 percent more likely to own and use a net compared with those 30 years and above (OR = 1.17, p = 0.021). Similar finding on age was evident for 'own a net but do not use' with about 30 percent of the respondents aged 25 to 29 years who owned a net but do not use (OR = 1.30, p = 0.039). A positive and significant association was evident with educational attainment for both categories. 'Ownership and use of net' increases with educational attainment. While respondents with only Primary education are about 45 percent likely to own and use a net (OR = 1.45, p < 0.0001), their counterparts with Secondary education are about 77 percent more likely to own and use a net (OR = 1.77, p < 0.0001) with those with higher education are most likely to own and use a net compared with their counterparts who do not have any formal education i.e. reference category (OR = 2.64, p < 0.0001). In this case, educational attainment is a protective effect for malaria. Caregivers in North East are significantly associated with ownership and use of a net (OR = 2.04, p < 0.0001). However, effect of Geopolitical zones was not significant for 'own a net but do not use'.

Misconception about causes and prevention of malaria are not significantly associated with 'ownership of net but did not use', p > 0.05. Findings also revealed that some caregivers with higher education are likely to own a net but did not use.

## Discussion

The findings of this survey have shown that misconceptions about the cause of malaria, its prevention and control by the care-givers of under-five Nigerian children have the potential to adversely influence the utilization of insecticide treated nets by the children. This observation is not novel. The influence of perception and knowledge about malaria on the attitude and practice of community members is widely acknowledged. The result of this study has reinforced similar observations by others [17,18] Nganda *et al *using knowledge scores (including knowledge of risks and consequences of malaria in pregnancy) showed that knowledge of malaria predicted ITN use, but not the use of intermittent preventive treatment [17]. Another African study involving pregnant women showed that the perception that nets could not prevent malaria was associated with non-ownership and non-use of ITN [18].

Several other studies including this have shown that poor perception and knowledge of malaria or its control is common among people living in areas where malaria is endemic and these have been associated with poor utilisation of malaria control services. Other studies have enumerated several misperceptions people hold that may inhibit appropriate preventive and therapeutic actions. For example, studies in Tanzania and Ghana have reported that malaria is perceived to result from excessive heat and overwork [19,20].

Another notable observation in this study is that while misconception about the cause and prevention of malaria was significantly associated with non-use of ITNs, we observed no association between knowledge of symptoms or risk of malaria and the use of ITNs. Findings from a similar study from another West African country showed no significant association between knowledge and the use of ITNs suggesting that relationship between knowledge and health action may be influenced by other biosocial factors [21].

While our aim in this report has been to highlight the significance of misconception as a predictor of non-use of mosquito nets, it is important to state that we identified other factors, which have also been reported by others as determinants of ownership and use nets. Such factors as education and socio-economic status have been severally reported to influence the use of ITNs and other malaria control services. This study observed that educational status and residence in certain geo-political areas of the country influenced the use of ITN, and corroborates the findings of two other Nigerian studies [22,23]. Dike *et al *working in south-east Nigeria observed that likelihood that an individual to own or use a bed net increased as the number the person's years of education increased [22]. Another Nigerian studied showed that the use of ITN by under-five Nigerian children was four times more likely in the coastal south (Niger Delta) than the arid south (Sahel Savannah) [23]. It is likely that such other covariates as socio-economic status and cultural differences (and not merely differences in geographic locations) may have contributed to the observed differences. Unfortunately neither that study nor ours undertook sufficient ethnographic evaluation to have elicited the likely effects of these qualitative covariates. A study of Kenyan homesteads showed that women from least poor homesteads were more likely to sleep under ITN than most poor [24].

This study has drawn attention to the need to identify and correct misconceptions about malaria. This would significantly enhance ongoing efforts to increase and sustain ITN use in Nigeria. The Global Fund to Fight AIDS, Tuberculosis and Malaria (GFATM) and other global initiatives have recently provided funding to country programmes to improve the scale-up of anti-malaria interventions particularly for the universal coverage of nets. In a bid to beat the 2010 deadline for achieving Roll Malaria target, massive campaigns to distribute several millions more bed nets has been initiated with support from several development partners. While these distribution campaigns are likely to rapidly increase ownership to high levels in a short time, it is needful to identify and remedy factors that could militate against the effective utilisation of these nets. People in different societies hold a variety of beliefs about the cause and transmission of malaria that vary according to cultural, educational, and economic factors, and have direct consequences for both preventive and treatment-seeking behaviour as well as for other activities to control malaria.

Studies in south-eastern and south-western Nigeria have identified such misconceptions about malaria as excessive heat, over-work, sunlight, excessive sex, noise and witchcraft as causes of malaria [9,10]. Failure to implement appropriate communication strategies to specifically address these and other misconceptions would result in their persistence with potential adverse consequences on the utilization of ITNs and other malaria control services. The protective efficacy of ITN against malaria infections is no longer in doubt but there is evidence that the community effectiveness of this intervention can be compromised by programmatic deficiencies [25].

## Conclusions

While the influence of misconception on health-seeking behaviour and practice may be widely acknowledged, the extent to which this evidence is applied to the design and implementation of public health interventions is far from being adequate. This paper has availed us opportunity to discern determinants of net use, for the 3 level outcome variable on ownership and use of ITN. Misconception about prevention and causes of malaria are found to be significantly associated with own and use of a net. Respondents who have some misconception are less likely to use a net even though they may have the net. The programmatic implication from this finding is that, behaviour change communication messages should address issues on misconception about causes of malaria and modes of its prevention.

## Competing interests

The authors declare that they have no competing interests.

## Authors' contributions

EDA, SBA and MMM analyzed the data and drafted the paper; all other authors contributed to the preparation of this paper. All authors read and approved the final manuscript.
